# Potential Impact of COVID-19 on Female Reproductive Health

**DOI:** 10.5935/1518-0557.20220019

**Published:** 2023

**Authors:** Nisha Kumari, Neha Kumari, Sumit Mishra

**Affiliations:** 1 Department of Chemistry, Birla Institute of Technology, Mesra, Ranchi-835215, Jharkhand, India

**Keywords:** COVID-19, female reproductive, angiotensin converting enzyme

## Abstract

COVID-19 has emerged as the biggest pandemic of the world of all times. Its death toll is rising globally. COVID-19 mostly affects the lungs because the virus enters the host cells via the receptor for the ACE2 enzyme, which is also present in other organs of the human body. ACE2 plays the main role in the degradation of Ang II, resulting in the formation of angiotensin 1-7 (Ang 1-7) which maintains the level of Ang II. This communication gives an assessment of reproductive system functioning and its effects by the COVID-19 exposure. It is important to maintain the wellbeing for healthy nourishment of the fetus and safe delivery along with post health issues. ACE2 enzyme metabolism is expressed in the female reproductive system, and it may be potential target of COVID-19 exposure.

## INTRODUCTION

Pneumonia cases of unknown etiology in the Wuhan city of China were reported in December, 2019 to the world health organization (WHO) and spread rapidly throughout the world (Wastnedge *et al*., 2021). Literature search shows that COVID-19 not only affects the female fertility but also damages the female reproductive functions (Jing *et al*., 2020; Li *et al*., 2020).

**Figure 1:**
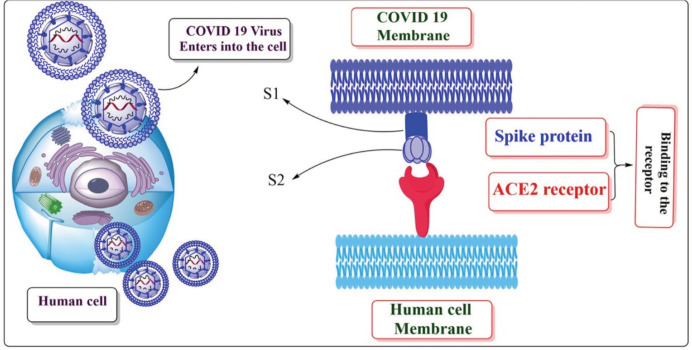
Systematic diagram presenting the spike protein of COVID-19 virus binding to the ACE2 (Angiotensin converting enzyme II) receptor and penetrating the targeted host cell.

COVID-19 enters the target host cell by binding to the angiotensin converting enzyme II receptor, which is used as a primary receptor binding to the host cell (Matsuyama *et al*., 2010; Zhou *et al*., 2015), and the expression of angiotensin converting enzyme II is regulated in the host cells (Jing *et al*., 2020), as shown in the Figure 1. Angiotensin converting enzyme has been evaluated in the female reproductive system such as the ovaries, uterus, vagina and placenta and also in the other parts of the human body, including the respiratory tract, kidneys, heart and gastrointestinal system (Lippi *et al*., 2020). Notably, the angiotensin converting enzyme plays a regulatory role in reproduction (Fu *et al*., 2020) and may be disturbed by attacking ovarian tissue or destroying endometrial epithelial cells (Li *et al*., 2021). For COVID-19, Basigin is also considering as one of the most important receptors expressed in the uterus, in the stroma, and also in the ovary granulosa cells (Chen *et al*., 2010; Mahdian *et al*., 2020).

### Female reproductive system

COVID-19 may affect the female reproductive system through invading the target cells, by binding to ACE2 (angiotensin-converting enzyme II). ACE2 usually maintains the levels of angiotensin II and angiotensin (1-7) to apply its special action, which is widely expressed in the vagina, uterus, ovaries and placenta. The reason of female fertility is the ovarian reserve, which could affect fecundity by decreasing egg quality when diminished (Steiner *et al*., 2017). Hence, the function of ovarian reserve for the impact of COVID-19 is the primary observation indicator on female fertility. Additionally, to explore heterogeneity we must include the type of fertility decline, age, the subgroup analyses and sensitivity studies are conducted. It is believed that fertility approximately starts at the age of thirteen and at the age of forty-nine the women begins to become infertile (Jensen *et al*., 2018). Female fertility declines naturally with ageing. At the age of thirty to thirty-five the decline in fertility is slow and steady. But, because of reduction in the ovarian reserve and oocyte quality, the decline increases after the age of thirty-five years (Ahmed *et al*., 2020).

### ACE2 expressed in the ovary

In ovaries from both reproductive-age women and postmenopausal women, ACE2 mRNA transcripts were detected (Reis *et al*., 2011). From the Gene Cards, we analyzed the ACE2 data and came to know that ACE2 is extremely present in the ovary. Therefore, the ovary might be the potential target of COVID 19 (Jing *et al*., 2020).

The key enzyme ACE2 in the axis plays a very important synergistic role in maintaining the levels of both angiotensin II (Ang II) and angiotensin [Ang (1-7)]. The function of Ang II induces steroid secretion (Hayashi *et al*., 2003), oocyte maturation (Giometti *et al*., 2005; Stefanello *et al*., 2006; Yoshimura *et al*., 1992), facilitates follicle development (Ferreira *et al*., 2011), influences ovulation (Acosta *et al*., 2000; Ferreira *et al*., 2007; Guo *et al*., 2012; Kuji *et al*., 1996; Kuo *et al*., 1991), and also maintains corpus luteum progression (Sugino *et al*., 2005). The function of Angiotensin (1-7) is mainly to promote the production of both progesterone and estradiol (Costa *et al*., 2003), and ovulation enhancement (Tonellotto dos Santos *et al*., 2012; Viana *et al*., 2011).

The most common endocrinopathy that is polycystic ovary syndrome, mainly affects reproductive-aged women, with a frequency which may exceed 10 to 15%, according to the population studied and the criteria used for the diagnostic. COVID-19 has certain factors known to have direct associations with PCOS, including hyper-inflammation, ethnicity predisposition, hyperandrogenism, and very low vitamin-D levels. Furthermore, there is markedly high frequency of female patient populations with multiple cardio-metabolic conditions, including obesity, hypertension and type 2 diabetes, which may increase the risk of adverse COVID-19 consequences.

### ACE2 expressed in uterus and vagina

In the uterus of both human (Vaz-Silva *et al*., 2009) and rats (Brosnihan *et al*., 2012), there is ACE2 mRNA, and the presence of ACE2 has been confirmed after analyzing the report from the Swedish based program and Human Genes database (Jing *et al*., 2020). In the regeneration of endometrium, vascular bed, menstruation initiation through spiral artery vasoconstriction, Angiotensin II plays a double role (Ahmed *et al.*, 1995). Furthermore, Ang II enhances the endometrial fibrosis and also increases the stroma cells and proliferation of uterus epithelium (Shan *et al*., 2014; 2015). In the endometrium, Angiotensin II functions for normal menarche, and sometimes alteration and dysfunctional uterine bleeding related with hyperplastic endometria (Li & Ahmed, 1996).

### ACE2 expressed in pregnancy

**Figure 2:**
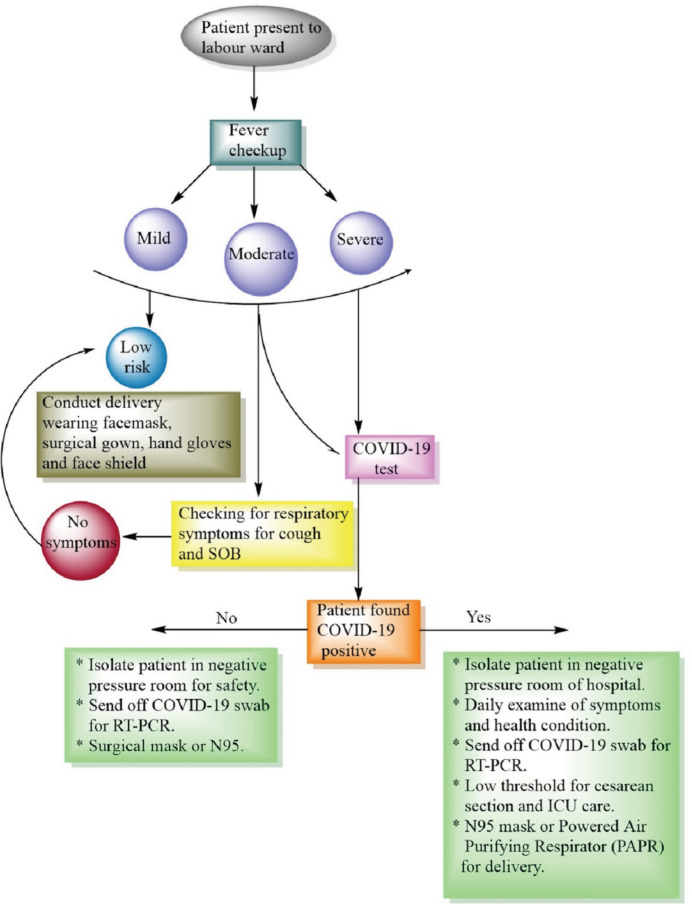
Schematic representation demonstrating the risk of the patients presenting to the delivery ward.

All Angiotensin II, angiotensin (1-7) and ACE2 function mainly during pregnancy through regulating fetus formation and regulating blood pressure (Hering *et al*., 2010). Both ACE2 and angiotensin (1-7) act as a paracrine regulator in the early and late events of pregnancy (Neves *et al*., 2008). To control and balance the hydro-salinity and blood pressure of a pregnant women, ACE2 hydrolyzes angiotensin II into angiotensin (1-7), and angiotensin I hydrolyzes into angiotensin (1-9) and changes to angiotensin (1-7) (Pringle *et al*., 2011). To the pregnant women and fetuses, COVID-19 infection poses a high threat and causes fetal distress, premature birth and rupture of fetal membranes and caesarean section (Chen *et al*., 2020a;b; Zhu *et al*., 2020). Women brought to the labor ward should be checked according to the symptoms of COVID 19 and must be divided into separate ward based on the condition of their COVID report shown in the Figure 2.

## CONCLUSION

COVID-19 may infect the female reproductive system through the expression of ACE2 enzyme present in the different parts of the human body, which may cause fetal distress, menstrual disorder and infertility. Pregnant women should take the suitable measures and precautions during routine check-ups and visits to the hospital and labor ward.

## References

[r1] Acosta TJ, Ozawa T, Kobayashi S, Hayashi K, Ohtani M, Kraetzl WD, Sato K, Schams D, Miyamoto A (2000). Periovulatory changes in the local release of vasoactive
peptides, prostaglandin f(2alpha), and steroid hormones from bovine mature
follicles in vivo. Biol Reprod.

[r2] Ahmed A, Li XF, Shams M, Gregory J, Rollason T, Barnes NM, Newton JR (1995). Localization of the angiotensin II and its receptor subtype
expression in human endometrium and identification of a novel high-affinity
angiotensin II binding site. J Clin Invest.

[r3] Ahmed TA, Ahmed SM, El-Gammal Z, Shouman S, Ahmed A, Mansour R, El-Badri N (2020). Oocyte Aging: The Role of Cellular and Environmental Factors and
Impact on Female Fertility. Adv Exp Med Biol.

[r4] Brosnihan KB, Bharadwaj MS, Yamaleyeva LM, Neves LA (2012). Decidualized pseudopregnant rat uterus shows marked reduction in
Ang II and Ang-(1-7) levels. Placenta.

[r5] Chen L, Bi J, Nakai M, Bunick D, Couse JF, Korach KS, Nowak RA (2010). Expression of basigin in reproductive tissues of estrogen
receptor-{alpha} or -{beta} null mice. Reproduction.

[r6] Chen H, Guo J, Wang C, Luo F, Yu X, Zhang W, Li J, Zhao D, Xu D, Gong Q, Liao J, Yang H, Hou W, Zhang Y (2020a). Clinical characteristics and intrauterine vertical transmission
potential of COVID-19 infection in nine pregnant women: a retrospective
review of medical records. Lancet.

[r7] Chen L, Li Q, Zheng D, Jiang H, Wei Y, Zou L, Feng L, Xiong G, Sun G, Wang H, Zhao Y, Qiao J (2020b). Clinical Characteristics of Pregnant Women with Covid-19 in
Wuhan, China. N Engl J Med.

[r8] Costa AP, Fagundes-Moura CR, Pereira VM, Silva LF, Vieira MA, Santos RA, Dos Reis AM (2003). Angiotensin-(1-7): a novel peptide in the ovary. Endocrinology.

[r9] Ferreira R, Oliveira JF, Fernandes R, Moraes JF, Gonçalves PB (2007). The role of angiotensin II in the early stages of bovine
ovulation. Reproduction.

[r10] Ferreira R, Gasperin B, Rovani M, Santos J, Barreta M, Bohrer R, Price C, Gonçalves PB (2011). Angiotensin II signaling promotes follicle growth and dominance
in cattle. Endocrinology.

[r11] Fu J, Zhou B, Zhang L, Balaji KS, Wei C, Liu X, Chen H, Peng J, Fu J (2020). Expressions and significances of the angiotensin-converting
enzyme 2 gene, the receptor of SARS-CoV-2 for COVID-19. Mol Biol Rep.

[r12] Giometti IC, Bertagnolli AC, Ornes RC, da Costa LF, Carambula SF, Reis AM, de Oliveira JF, Emanuelli IP, Gonçalves PB (2005). Angiotensin II reverses the inhibitory action produced by theca
cells on bovine oocyte nuclear maturation. Theriogenology.

[r13] Guo B, Zhang XM, Li SJ, Tian XC, Wang ST, Li DD, Liu DF, Yue ZP (2012). Expression and regulation of Ang-2 in murine ovaries during
sexual maturation and development of corpus luteum. Mol Biol (Mosk).

[r14] Hayashi KG, Acosta TJ, Tetsuka M, Berisha B, Matsui M, Schams D, Ohtani M, Miyamoto A (2003). Involvement of angiopoietin-tie system in bovine follicular
development and atresia: messenger RNA expression in theca interna and
effect on steroid secretion. Biol Reprod.

[r15] Hering L, Herse F, Geusens N, Verlohren S, Wenzel K, Staff AC, Brosnihan KB, Huppertz B, Luft FC, Muller DN, Pijnenborg R, Cartwright JE, Dechend R (2010). Effects of circulating and local uteroplacental angiotensin II in
rat pregnancy. Hypertension.

[r16] Jensen RE, Martins N, Parks MM (2018). Public Perception of Female Fertility: Initial Fertility, Peak
Fertility, and Age-Related Infertility Among U.S. Adults. Arch Sex Behav.

[r17] Jing Y, Run-Qian L, Hao-Ran W, Hao-Ran C, Ya-Bin L, Yang G, Fei C (2020). Potential influence of COVID-19/ACE2 on the female reproductive
system. Mol Hum Reprod.

[r18] Kuji N, Sueoka K, Miyazaki T, Tanaka M, Oda T, Kobayashi T, Yoshimura Y (1996). Involvement of angiotensin II in the process of
gonadotropin-induced ovulation in rabbits. Biol Reprod.

[r19] Kuo TC, Endo K, Dharmarajan AM, Miyazaki T, Atlas SJ, Wallach EE (1991). Direct effect of angiotensin II on in-vitro perfused rabbit
ovary. J Reprod Fertil.

[r20] Li F, Lu H, Zhang Q, Li X, Wang T, Liu Q, Yang Q, Qiang L (2021). Impact of COVID-19 on female fertility: a systematic review and
meta-analysis protocol. BMJ Open.

[r21] Li R, Yin T, Fang F, Li Q, Chen J, Wang Y, Hao Y, Wu G, Duan P, Wang Y, Cheng D, Zhou Q, Zafar MI, Xiong C, Li H, Yang J, Qiao J (2020). Potential risks of SARS-CoV-2 infection on reproductive
health. Reprod Biomed Online.

[r22] Li XF, Ahmed A (1996). Expression of angiotensin II and its receptor subtypes in
endometrial hyperplasia: a possible role in dysfunctional
menstruation. Lab Invest.

[r23] Lippi G, Lavie CJ, Henry BM, Sanchis-Gomar F (2020). Do genetic polymorphisms in angiotensin converting enzyme 2
(ACE2) gene play a role in coronavirus disease 2019
(COVID-19)?. Clin Chem Lab Med.

[r24] Mahdian S, Shahhoseini M, Moini A (2020). COVID-19 Mediated by Basigin Can Affect Male and Female
Fertility. Int J Fertil Steril.

[r25] Matsuyama S, Nagata N, Shirato K, Kawase M, Takeda M, Taguchi F (2010). Efficient activation of the severe acute respiratory syndrome
coronavirus spike protein by the transmembrane protease
TMPRSS2. J Virol.

[r26] Neves LA, Stovall K, Joyner J, Valdés G, Gallagher PE, Ferrario CM, Merrill DC, Brosnihan KB (2008). ACE2 and ANG-(1-7) in the rat uterus during early and late
gestation. Am J Physiol Regul Integr Comp Physiol.

[r27] Pringle KG, Tadros MA, Callister RJ, Lumbers ER (2011). The expression and localization of the human placental
prorenin/renin-angiotensin system throughout pregnancy: roles in trophoblast
invasion and angiogenesis?. Placenta.

[r28] Reis FM, Bouissou DR, Pereira VM, Camargos AF, dos Reis AM, Santos RA (2011). Angiotensin-(1-7), its receptor Mas, and the
angiotensin-converting enzyme type 2 are expressed in the human
ovary. Fertil Steril.

[r29] Shan T, Shang W, Zhang L, Zhao C, Chen W, Zhang Y, Li G (2015). Effect of angiotensin-(1-7) and angiotensin II on the
proliferation and activation of human endometrial stromal cells in
vitro. Int J Clin Exp Pathol.

[r30] Shan T, Zhang L, Zhao C, Chen W, Zhang Y, Li G (2014). Angiotensin-(1-7) and angiotensin II induce the
transdifferentiation of human endometrial epithelial cells in
vitro. Mol Med Rep.

[r31] Stefanello JR, Barreta MH, Porciuncula PM, Arruda JN, Oliveira JF, Oliveira MA, Gonçalves PB (2006). Effect of angiotensin II with follicle cells and insulin-like
growth factor-I or insulin on bovine oocyte maturation and embryo
development. Theriogenology.

[r32] Steiner AZ, Pritchard D, Stanczyk FZ, Kesner JS, Meadows JW, Herring AH, Baird DD (2017). Association Between Biomarkers of Ovarian Reserve and Infertility
Among Older Women of Reproductive Age. JAMA.

[r33] Sugino N, Suzuki T, Sakata A, Miwa I, Asada H, Taketani T, Yamagata Y, Tamura H (2005). Angiogenesis in the human corpus luteum: changes in expression of
angiopoietins in the corpus luteum throughout the menstrual cycle and in
early pregnancy. J Clin Endocrinol Metab.

[r34] Tonellotto dos Santos J, Ferreira R, Gasperin BG, Siqueira LC, de Oliveira JF, Santos RA, Reis AM, Gonçalves PB (2012). Molecular characterization and regulation of the
angiotensin-converting enzyme type 2/angiotensin-(1-7)/MAS receptor axis
during the ovulation process in cattle. J Renin Angiotensin Aldosterone Syst.

[r35] Vaz-Silva J, Carneiro MM, Ferreira MC, Pinheiro SV, Silva DA, Silva-Filho AL, Witz CA, Reis AM, Santos RA, Reis FM (2009). The vasoactive peptide angiotensin-(1-7), its receptor Mas and
the angiotensin-converting enzyme type 2 are expressed in the human
endometrium. Reprod Sci.

[r36] Viana GE, Pereira VM, Honorato-Sampaio K, Oliveira CA, Santos RA, Reis AM (2011). Angiotensin-(1-7) induces ovulation and steroidogenesis in
perfused rabbit ovaries. Exp Physiol.

[r37] Wastnedge EAN, Reynolds RM, van Boeckel SR, Stock SJ, Denison FC, Maybin JA, Critchley HOD (2021). Pregnancy and COVID-19. Physiol Rev.

[r38] Yoshimura Y, Karube M, Koyama N, Shiokawa S, Nanno T, Nakamura Y (1992). Angiotensin II directly induces follicle rupture and oocyte
maturation in the rabbit. FEBS Lett.

[r39] Zhou Y, Vedantham P, Lu K, Agudelo J, Carrion R Jr, Nunneley JW, Barnard D, Pöhlmann S, McKerrow JH, Renslo AR, Simmons G (2015). Protease inhibitors targeting coronavirus and filovirus
entry. Antiviral Res.

[r40] Zhu H, Wang L, Fang C, Peng S, Zhang L, Chang G, Xia S, Zhou W (2020). Clinical analysis of 10 neonates born to mothers with 2019-nCoV
pneumonia. Transl Pediatr.

